# Myocardial Oedema as a Consequence of Viral Infection and Persistence—A Narrative Review with Focus on COVID-19 and Post COVID Sequelae

**DOI:** 10.3390/v16010121

**Published:** 2024-01-14

**Authors:** Noel G. Panagiotides, Michael Poledniczek, Martin Andreas, Martin Hülsmann, Alfred A. Kocher, Christoph W. Kopp, Aleksandra Piechota-Polanczyk, Annika Weidenhammer, Noemi Pavo, Patricia P. Wadowski

**Affiliations:** 1 Division of Cardiology, Department of Internal Medicine II, Medical University of Vienna, 1090 Vienna, Austria; noel.panagiotides@meduniwien.ac.at (N.G.P.); michael.poledniczek@meduniwien.ac.at (M.P.); martin.huelsmann@meduniwien.ac.at (M.H.); annika.weidenhammer@meduniwien.ac.at (A.W.); noemi.pavo@meduniwien.ac.at (N.P.); 2 Division of Angiology, Department of Internal Medicine II, Medical University of Vienna, 1090 Vienna, Austria; christoph.kopp@meduniwien.ac.at; 3 Department of Cardiac Surgery, Medical University of Vienna, 1090 Vienna, Austria; martin.andreas@meduniwien.ac.at (M.A.); alfred.kocher@meduniwien.ac.at (A.A.K.); 4 Department of Cell Cultures and Genomic Analysis, Medical University of Lodz, 90-752 Łódź, Poland; aleksandra.piechota-polanczyk@umed.lodz.pl

**Keywords:** myocardial oedema, glycocalyx, coronavirus disease 2019, microcirculation, platelets

## Abstract

Microvascular integrity is a critical factor in myocardial fluid homeostasis. The subtle equilibrium between capillary filtration and lymphatic fluid removal is disturbed during pathological processes leading to inflammation, but also in hypoxia or due to alterations in vascular perfusion and coagulability. The degradation of the glycocalyx as the main component of the endothelial filtration barrier as well as pericyte disintegration results in the accumulation of interstitial and intracellular water. Moreover, lymphatic dysfunction evokes an increase in metabolic waste products, cytokines and inflammatory cells in the interstitial space contributing to myocardial oedema formation. This leads to myocardial stiffness and impaired contractility, eventually resulting in cardiomyocyte apoptosis, myocardial remodelling and fibrosis. The following article reviews pathophysiological inflammatory processes leading to myocardial oedema including myocarditis, ischaemia-reperfusion injury and viral infections with a special focus on the pathomechanisms evoked by severe acute respiratory syndrome coronavirus 2 (SARS-CoV-2) infection. In addition, clinical implications including potential long-term effects due to viral persistence (long COVID), as well as treatment options, are discussed.

## 1. Introduction

Myocardial fluid homeostasis is based on a complex interaction between microvascular filtration and absorption, interstitial hydration, as well as water uptake by cardiomyocytes, and lymphatic removal [1]. Pathological conditions like ischaemia, ischaemia-reperfusion injury, inflammation, and hypertension disturb the subtle equilibrium and dysregulate myocardial fluid dynamics [2]. As a consequence, tissue oedema, characterized by the accumulation of water in interstitial and intracellular compartments, occurs and results in cardiomyocyte injury, dysfunction, and subsequent cardiac remodelling [3,4,5,6].

Myocardial oedema (MO) has been identified in various cardiac diseases including heart failure (HF) [1], ischaemia-reperfusion injury [7], and myocarditis [8,9]. MO develops not only due to disturbances to the microvascular barrier leading to increased endothelial permeability through glycocalyx degradation and pericyte detachment but also due to changes in the composition of the myocardial extracellular matrix (mECM), the cardiac lymphatic system and cardiomyocyte homoeostasis [1]. Myocardial cells play a critical role in fluid homeostasis regulation through the control of ion pumps and membrane-bound proteins [10].

The myocardium is considered to be one of the most vulnerable tissues for oedema formation due to high metabolic demand, low oxygen extraction reserve [11] and autoregulation of blood flow, which relies on close proximity between cardiomyocytes and endothelial cells [12]. Consequently, MO may contribute to myocyte ischaemia by expanding the interstitial space and consecutively increasing distances for oxygen transport [13].

The mECM is integral for the physiologic function of the myocardium [14,15], with its primary components being collagen subtypes I and III, which provide stability and tensile strength [16]. Beyond mechanical support, the mECM is actively involved in signal transduction, regulating cellular differentiation, growth and survival [17].

The disruption of the balance between mECM degeneration through enzymatic proteolysis by matrix-metalloproteinases and its inhibition by tissue inhibitors of metalloproteinases (TIMPs) results in increased collagen deposition and manifests as myocardial fibrosis [14]. Degradation and remodelling of the mECM have been observed in acute myocarditis [18] and in chronic heart failure [4,19], with MO playing an active role in promoting these processes [4,20]. MO can facilitate myocardial fibrosis development by increasing mRNA levels of collagen types I and III as well as prolyl 4-hydroxylase [20]. Increased collagen deposition is associated with impaired cardiac contractility, greater left ventricular stiffness and HF [21,22].

Endothelial dysfunction in combination with the breakdown of the endothelial protective layer, i.e., the glycocalyx, can also be induced by SARS-CoV-2 infection. This contributes to MO formation through heightened inflammation and generates a pro-thrombotic state via activation of platelets and the coagulation cascade [23,24,25,26,27].

Furthermore, the interaction of pathological processes initiated or exacerbated by MO, including inflammation, endothelial cell dysfunction, release of reactive oxygen species and proinflammatory cytokine signalling, contribute to a vicious circle of immunothrombosis, ultimately leading to tissue necrosis, fibrosis and organ failure [28,29].

## 2. Physiological Background of Myocardial Fluid Filtration

### 2.1. Starling Forces and Microvascular Fluid Filtration

A fundamental framework for understanding the forces regulating microvascular fluid exchange, homeostasis and its dysregulation leading to MO can best be described by the Starling principle [28] and the revised Starling equation (Table 1) [29]. The rate of fluid filtration (J_V_) per surface filtration area (S) is dependent on the capillary hydraulic conductivity (L_P_), the difference in capillary and interstitial hydrostatic pressure (delta P) and the difference in osmotic pressure across the membrane derived from plasma proteins (delta Π) [29]. This rate is strongly influenced by the permeability of the endothelial barrier to various solutes, primarily, which are expressed by the protein reflection coefficient σ [30]. 

Essentially, the rate of volume filtration per endothelial area is dependent on membrane permeability, differences in intracapillary and interstitial hydrostatic pressure, and variations in intracapillary and interstitial colloid osmotic pressure.

P_C_ has a major influence on net fluid flow [2], while P_I_, which approximates arterial pressure during systole, is its main opposing force. Therefore, myocardial fluid exchange likely takes place predominantly during diastole [2,36]. Π_C_ is provided by plasma proteins unable to surpass the endothelial barrier and acts as a major opposing force to P_C_. Alterations of Π_C,_ for example, during hypoalbuminaemia, trigger interstitial fluid extravasation [43]. Furthermore, the glycocalyx as part of the endothelial barrier, not only binds certain proteins such as von Willebrand factor (vWF) or coagulation factor IX, but also regulates the passage of molecular substances such as fibrinogen or albumin based on their charge, thereby mediating fluid filtration via σ and Π_G_ [44,45,46].

A key factor in myocardial fluid homeostasis and preventing interstitial fluid accumulation is the removal of filtered fluid via lymphatic fluid drainage [1,47]. Although myocardial fluid exchange is suspected to occur mainly on the venular side of the capillary bed [48], it is important to recognize that most tissues greatly rely on lymphatic fluid removal rather than venous capillary absorption for interstitial fluid removal and tissue fluid balance [29]. This becomes evident as impaired lymphatic drainage is commonly accompanied by oedema formation [43]. Therefore, the accumulation of interstitial fluid, manifested as oedema, is the result of fluid filtration volume exceeding the lymphatic fluid drainage.

Alteration of Starling forces can lead to enhanced fluid filtration and result in MO [29]. Fluid accumulation in the interstitium occurs when the forces driving the fluid out of the vessel are greater than the opposing forces responsible for intravascular fluid retention. Factors favouring fluid filtration are high L_P_, high S, high P_C,_ high Π_G,_ low P_I,_ low Π_C, and_ low σ [29]. Increased capillary pressure can result from conditions such as acute or chronic arterial hypertension, pulmonary hypertension, right ventricular failure, fluid overload in decompensated heart failure or venous obstruction [20,39,43,49,50]. Reduced colloid osmotic pressure typically results from hypoalbuminaemia, which can be attributed to malnutrition, malabsorption, nephrotic syndrome or hepatic failure [43], leading to greater fluid flow and interstitial oedema [6]. This is especially relevant in cardiac surgery and shock management, where crystalloid coronary perfusion and high levels of fluid resuscitation worsen prognosis [2,51,52]. Furthermore, inflammation increases capillary recruitment and leads to vasodilation, which substantially enhances fluid filtration by increasing P_C_, capillary permeability, and blood flow and reducing the colloid osmotic pressure [29]. In addition, inflammation may cause a reduction in P_I_ via changes in the connective tissue and extracellular matrix [53].

### 2.2. The Glycocalyx as Key Regulator of the Endothelial Barrier

The endothelial surface layer (ESL) is a network made up of proteoglycans, glycoproteins and glycosaminoglycans, i.e., the glycocalyx, including syndecan-1, glypican, and hyaluronan, in conjunction with associated plasma proteins and soluble glycosaminoglycans [44,54]. The extension of the glycocalyx into the luminal surface of blood vessels varies depending on vessel size, and it projects into gaps between cells and fenestrations [44,54,55,56,57,58]. The glycocalyx exhibits constant change and is in equilibrium between continuous production and shedding due to enzymes or mechanical forces [44,59]. 

Functionally, the glycocalyx plays a vital role in the proper functioning of small blood vessels and serves critical vasoprotective functions [44]. It physically shields the underlying endothelium and limits immune cell interaction with the vessel walls [44,58,60]. It also regulates endothelial inflammation by capturing cytokines and restricting access to surface receptors [44,58,60]. The glycocalyx is negatively charged and contributes to the endothelial barrier by regulating plasma flow and passage of differently charged molecules [58,61]. In the context of the Frank–Starling equation, the glycocalyx and the subglycocalyx space exert opposing forces on volume filtration influencing permeability (L_P_, σ), and providing an opposing colloid osmotic pressure (Π_G_) against the intracapillary colloid osmotic pressure. This function results from anionic sites that repel negatively charged substances and cells, such as red blood cells, while facilitating the passage of positively charged proteins and molecules [45,61,62,63,64]. The removal of glycocalyx-binding proteins and further components increases conductivity adding to microvascular permeability [65,66,67,68,69,70]. Moreover, the glycocalyx regulates microvascular flow via transmission of shear-stress and nitric oxide (NO) production [71,72]. Furthermore, it protects against oxidative stress and endothelial dysfunction by binding antioxidant enzymes like superoxide dismutase to counteract oxygen radicals [73]. The intact glycocalyx also prevents platelet activation and thrombus formation [44,74] by binding and interacting with anticoagulant mediators such as antithrombin III [74] or heparin cofactor II [75].

## 3. Pathophysiology

### 3.1. Glycocalyx Disintegration

Glycocalyx disruption with concomitant breakdown of the endothelial barrier is described in many critical illnesses and can mostly be attributed to inflammation [76]. The greater exposure to the endothelial surface enables immune cells and harmful agents like proteases and reactive oxygen species (ROS) to interact more readily with the endothelium [76].

The endothelial glycocalyx has a critical role in preventing MO formation [77]. Damage to the coronary glycocalyx results in myocardial fluid accumulation and swelling of the pericapillary interstitial space [77,78]. Glycocalyx alteration or neutralization of its negative charge appears to affect its barrier function leading to enhanced permeability [61,79]. Experimental glycocalyx degradation was found to elevate capillary permeability [66], ultimately leading to myocardial oedema formation [77]. In vivo, alterations of the glycocalyx and microvascular permeability changes have been linked to conditions such as hypertension [49] and hypoproteinaemia [80], sepsis [81], viral infections including SARS-CoV-2 [23,24,25], inflammation and myocarditis [82]. Serious infections and systemic inflammation are known to increase microvascular permeability and induce cardiac dysfunction [83]. Viral infections enhance vascular permeability by upregulating glycocalyx-degrading enzymes such as sialidase and heparanase [84,85]. In sepsis, MO formation has been attributed to the shedding of negatively charged glycocalyx molecules [86].

Pro-inflammatory cytokines such as tumor necrosis factor-α (TNF-α) can disrupt the glycocalyx and increase permeability independent of leukocyte adhesion [87]. In addition, initial degradation of the glycocalyx eases leukocyte and platelet adhesion to endothelial cells [44,60]. Cytokines activate both leukocytes and endothelial cells, leading to the upregulation of adhesion molecules and facilitating the recruitment of inflammatory cells to sites of inflammation [88]. After the adhesion of leukocytes to the endothelium, the release of proteases and free radicals can exacerbate endothelial damage that can contribute to oedema formation [89,90,91,92]. Inflammatory stimuli also affect the endothelial barrier by targeting downstream signalling molecules that ultimately control intercellular junctions [93] (see also Section 3.2).

Hypoxia has been linked to increased permeability via glycocalyx disruption and MO formation [94]. Additionally, in the context of inflammation and atherosclerosis, oxidized low-density lipoprotein (LDL) has been shown to degrade the glycocalyx [95], increase microvascular permeability, [96,97] and facilitate adhesion of leucocytes, potentially enhancing atherosclerotic plaque development [98,99,100].

Moreover, damage to the endothelial surface layer can also be observed in the setting of inflammation related to ischaemia-reperfusion injury [101,102,103]. The generation of ROS triggers NLRP3 inflammasome activation leading to caspase-1-mediated pyroptosis [104,105,106].

Numerous reports suggest a major role for inflammation and hypoxia in the development of microvascular obstruction and dysfunction; this triggers not only chronic atherosclerotic processes but can lead to acute myocardial injury including infarction with potentially lethal complications [104,107,108,109,110]. Herein, the high presence of fibrin-rich micro-thrombi in myocardial capillaries, arterioles, as well as small muscular arteries, drives myocardial injury and tissue necrosis [111]. Following ischaemia endothelial protrusions, increased endothelial permeability and platelet–neutrophil aggregates can be observed, which, in turn, aggravate microvascular dysfunction by the release of vasoconstrictive and pro-inflammatory molecules including among others leukotrienes, thromboxane A2, platelet-derived growth factor, and ROS [108,112,113,114]. The accumulation of metabolites during hypoxia shifts the myocardial osmotic balance, while also exerting toxic effects on cardiomyocytes and endothelial cells [108,113]. The ensuing formation of MO, induced by increased vascular permeability and a shift in osmotic balance, further decreases tissue oxygen supply by compression of the microvasculature [108,112,115]. In addition to damage to the endothelial surface layer, microvascular dysfunction and obstruction are also aggravated by the effects of ROS on arteriole vasomotor function and promotion of thrombus formation—a pathomechanism, which may also apply to COVID-19 [104,111,116,117,118].

Ischaemia-related changes and chronic inflammation serve as potent triggers for proangiogenic factors such as vascular endothelial growth factor (VEGF) and hypoxia-inducible factor (HIF)-1 alpha [119,120,121,122]. VEGF can increase vascular permeability via SRC signalling, facilitating protein and cell migration for angiogenesis [93,123]. Aside from VEGF, matrix metalloproteinases (MMP) play a key role in angiogenesis, as they degrade the extracellular matrix [124]. Inflammatory conditions and exposure to inflammatory mediators, such as TNF-α, can enhance MMP expression and activity [125]. Importantly, MMP9 is associated with a greater risk of coronary atherosclerosis and cardiovascular events [126].

Cardiac natriuretic peptides have a significant impact on circulatory balance via their strong natriuretic and diuretic effects [127]. Natriuretic peptides like A-type natriuretic peptide (ANP) can also increase capillary permeability by increasing capillary filtration [128,129]. Experimental ANP administration caused degradation of the glycocalyx contributing to enhanced fluid filtration [130]. Besides ANP, vascular barrier function was also influenced by B- and C-type natriuretic peptides (BNP and CNP) leading to the shedding of glycocalyx components such as syndecan-1 and heparan sulphate [131]. NP-related shedding of glycocalyx components was accompanied by enhanced permeability and significant fluid extravasation [131,132]. Interestingly, the N-terminal prohormone of BNP (NT-proBNP) levels have been shown to be independently linked with in-hospital mortality of COVID-19 patients with pneumonia, but without HF [133]. Moreover, NT-proBNP serves as an efficient biomarker for identifying patients at risk of cardiac events, as demonstrated in patients with type 2 diabetes without preexisting cardiac disease [134].

### 3.2. Intercellular Junctions and Key Signalling Processes

Besides the glycocalyx, the endothelial barrier is upheld by intercellular junctions, i.e., tight and adherens junctions, which cover the intercellular gap between endothelial cells [58,61,93].

Tight junctions (TJs) consist of three main components: claudins, occludins and junction adhesion molecules [135]. They are attached to the cellular actin cytoskeleton via cingulin, which interacts with one of the zonula occluden (ZO) proteins (ZO-1, 2, or 3) [136]. 

TJs are controlled by Rho GTPases, kinases and phosphatases [93,137,138,139,140,141]. Adherens junctions (AJs) are also connected to the actin cytoskeleton via vascular endothelial-cadherin (VE-cadherin), a key protein in controlling and maintaining the endothelial barrier [142]. VE-cadherin also interacts with several kinases, phosphatases and other signalling molecules, which control its function and ultimately mediate VE-cadherin stability [93,143,144]. Herein, actin plays a decisive role in regulating the endothelial barrier [145]. Actin filaments attached to tight and adherens junction proteins can open intercellular gaps via contraction after a rise in cytosolic Ca^2+^ and activation of the myosin-light-chain kinase with concomitant inhibition of the myosin-light-chain-phosphatase through RhoA signalling [146]. This reorganization of actin filaments can be triggered by various inflammatory mediators [147,148,149]. The Rho-GTPase Ras-related C3 botulinum toxin substrate 1 (Rac1) is a signalling molecule that coordinates actin-binding proteins to stabilize intercellular junctions [150]. Under physiological conditions, Rac1 increases endothelial barrier function via signalling pathways involving sphingosine-1-phosphate (S1P) [151,152,153] or angiopoietin-1 (Ang-1) [93,154,155]. S1P stabilizes the glycocalyx by decreasing the activity of MMPs, hereby reducing the shedding of syndecan-1, chondroitinsulphate and heparan sulphate [156]. Signalling pathways involving guanine nucleotide exchange factors (GEFs) such as T-lymphoma invasion and metastasis-inducing protein 1 (Tiam1), guanine nucleotide exchange factor 2 (Vav2), and Trio Rho guanine nucleotide exchange factor (Trio) activate Rac1 [93,157,158]. These GEFs are regulated by cyclic adenosine monophosphate (cAMP) signalling, predominantly through guanine nucleotide exchange factor 3 (Epac1) dependent pathways as opposed to those initiated by protein kinase A (PKA) [159,160]. Elevated levels of cAMP enhance the endothelial barrier via activation of Rac1 and can protect against external barrier-compromising factors [157,161,162].

Inflammation disrupts the endothelial barrier by opening TJ and AJ, resulting in increased permeability, which facilitates the transport of fluids, soluble substances and cells [93,157,163,164,165,166,167]. There are two main factors contributing to this process: the phosphorylation of AJ components [144,168] and an imbalance in the signalling of Rac1 and RhoA in favour of RhoA, which enables actin-contraction on VE-cadherin and opening of junctions [93,146,169,170]. Both factors result in the endocytosis of VE-cadherin and the disassembly of junctions. In systemic inflammation, several inflammatory mediators are associated with AJ and TJ internalization and increased permeability [93]. For example, TNF-α, lipopolysaccharide (LPS) or thrombin can lead to a reduction in cAMP levels and deactivate Rac1 resulting in barrier breakdown and increased permeability [157,163,164,165,166]. TNF-α and LPS signalling involves activation of the metalloprotease a disintegrin and metalloproteinase 10 (ADAM10) [171]. Phosphorylation of VE-cadherin is facilitated by the activation of Src and other kinases [172].

### 3.3. Inflammation and Myocardial Oedema

MOs can occur as the consequence of acute or sub-acute events and herein as a response to inflammatory, ischemic or prothrombotic stimuli [10].

The impact of inflammation on the endothelial barrier and fluid filtration is profound, with the potential to multiply net fluid filtration several times (up to 17 fold) [29]. Moreover, pro-inflammatory cytokines can cause a systemic capillary leak syndrome, rarely resulting in myocardial involvement [173]. This is mostly due to interstitial oedema, which may progress to acute hypotension with cardiac shock and acute ventricular dysfunction [173]. Systemic capillary leak syndrome can be triggered by viral infections such as parvovirus B19, dengue virus or SARS-CoV2 [173,174,175]. The latter has been reported to be driven by the SARS-CoV-2 spike protein via the involvement of glycosaminoglycans, integrins, as well as the TGF-β signalling axis, altogether mediating endothelial dysfunction and extracellular matrix reorganization [175].

Importantly, the inflammation and oedema formation, amplify the activity of enzymes that degrade the mECM [176]. Usually beta1-integrin bound collagen fibrils to fibroblasts exert a compressive force countering interstitial expansion. However, during inflammation, these collagen fibrils can detach from fibroblasts, which greatly affects PI [29,53]. Inflammation and MO also result in impaired myocardial contraction and relaxation [52,177,178]. Moreover, MO disturbs ventricular filling through increased diastolic chamber stiffness [176]. The extent of MO has prognostic value in various myocardial disease entities including acute heart failure [50,179], myocardial infarction [180], aortic stenosis [181], pulmonary arterial hypertension [182], and myocarditis [183,184]. MO seen in HF can result from impaired myocardial contractility reducing lymph flow rate [185]. Additionally, it can be attributed to increased venous congestion affecting fluid dynamics [186] as well as inflammation and oxidative stress, which increase microvascular permeability [187,188].

In addition, pro-inflammatory cytokines disrupt the endothelial cells’ ability to protect against platelet activation through mechanisms like NO, prostacyclin production, and expression of CD39 (ecto-ADPase) [189,190,191,192]. Various cytokines such as TNF-α and IL-6 have been found to reduce the expression of CD39 in blood vessels, contributing to ADP-triggered platelet activation and enhanced interaction between platelets and white blood cells [193,194]. Moreover, IL-6 can induce angiogenesis via VEGF [195,196], leading to greater vascular permeability [170,197]. Further, TNF and interleukin-1 (IL-1) can facilitate the activation of endothelial cells, which can increase local blood flow via NO-mediated relaxation of vascular smooth muscle tone and increase leukocyte recruitment [198,199,200]. Inflammation effectively induces a collapse of the endothelial barrier and leads to increased movement of fluids, molecules and proteins into the interstitium [201], caused by increased capillary permeability, increased blood flow and reduced colloid osmotic pressure [29].

Cardiac pericytes, which interact with microvascular endothelium, are also implicated in myocardial remodelling associated with MO and concomitant inflammation [202]. Pericytes, located around endothelial cells in microvessels, are vital for capillary maintenance and functionality [203,204]. Pericytes regulate blood flow through an alteration of the capillary diameter in response to a series of vasoactive molecules [205,206]. Myocardial capillary pericytes also express angiotensin-converting enzyme 2 (ACE-2) [207], making them susceptible to SARS-CoV-2. Upon activation and inflammatory injury, pericytes may transdifferentiate into myofibroblast, which secrete mECM promoting myocardial fibrosis [208]. Additionally, pericytes can regulate the entry of immune cells [204] by either relaxing or widening gaps and allowing diapedesis of leukocytes [209,210]. Under certain circumstances, pericytes can become migratory, for example, after increased angiopoietin-2 signalling, hereby contributing to vascular dysfunction and increased permeability [211].

In short, inflammation promotes the degradation of the endothelial surface layer and triggers the detachment of pericytes, thereby compromising the microvascular barrier [77,82]. This causes excessive fluid filtration, formation of MO [1], and greater leukocyte adhesion and diapedesis [82,212,213].

### 3.4. Detection of Myocardial Oedema and Myocarditis

For the non-invasive evaluation of myocarditis, the updated Lake Louise Criteria are applied [214]. Therein, the combination of at least one finding indicative of MO and one finding of non-ischaemic myocardial injury is utilised to make a diagnosis of acute myocarditis with sufficient sensitivity and specificity [214]. Various cardiac magnetic resonance imaging (CMR) sequences are employed to evaluate MO, hyperaemia, capillary congestion or leakage, necrosis, and fibrosis [214].

MO is represented by prolonged T1 and T2 relaxation times due to increased extracellular fluid content. However, prolonged T1 times are also seen in fibrotic areas [215] and in the setting of acute decompensated heart failure with concurrent volume overload due to venous congestion [50]. However, unlike myocarditis, MO linked to heart failure-related congestion is relieved upon successful recompensation [50]. Similar to other tissues, hyperaemia and capillary leak are hallmarks of inflammation in the myocardium and are represented by early T1 mapping in CMR [216]. Contrast agents based on gadolinium, which mark the extracellular space expansion, show distinct myocardial uptake patterns in inflamed and non-inflamed areas of the myocardium [216]. Late gadolinium enhancement (LGE) in a non-subendocardial pattern is considered indicative of severe inflammation or fibrosis [214]. While less specific, ventricular dysfunction and pericardial involvement are supportive criteria for myocarditis [214]. Caution is advised in the context of myocardial infarction, as myocardial injury, including MO due to ischaemia and reperfusion injury may mimic some features also seen in myocarditis. However, these features typically exhibit different distribution patterns and may change during recovery [217,218].

MO can also occur due to Takotsubo syndrome, a condition that mimics acute coronary syndrome and can be triggered by stressful events, but also by viral infections like COVID-19 [115,219,220]. This syndrome manifests as contraction irregularities, including basal hyperkinesis and mid-ventricular or apical akinesis (apical ballooning) in the absence of significant coronary artery disease as well as transient left ventricular dysfunction and myocardial oedema with a circumferential and transmural distribution [221].

While less specific, the updated Lake Louise Criteria can also be utilised to identify chronic viral myocarditis [222]. However, it is worth noting that these criteria are not fully applicable to COVID-19 [223]. Also, CMR in acute viral myocarditis is yet unable to differentiate between myocarditis due to direct infection of the myocardium and secondary immune responses with cardiac involvement [222].

When routine cardiac workup and CMR are not sufficient to diagnose myocarditis, an endomyocardial biopsy (EMB) is recommended [224]. This has the advantage that subsequent analysis of EMB samples can not only help define the aetiology but also facilitate the decision on specific treatment options [225].

### 3.5. SARS-CoV-2 Infection

Recently, SARS-CoV-2 infection has become a major global burden with complications primarily impacting the respiratory and cardiovascular system [226,227]. The COVID-19 pandemic underscores the urgency of developing effective strategies against SARS-CoV-2, with small molecule inhibitors such as remdesivir, nirmatrelvir, and molnupiravir playing a crucial role alongside concepts of primary prevention (see also Section 4.1) [228]. Given the challenges of drug resistance and the emergence of new SARS-CoV-2 variants, there is a pressing need for innovative treatment approaches and more effective drugs [228]. Notably, ongoing efforts include the development of novel small-molecule anti-SARS-CoV-2 drugs like azvudine [229], VV116 [230], proxalutamide [231], FB2001 [232], WPV01 [233], pentarlandir [234], and cepharanthine [235].

**Figure 1:**
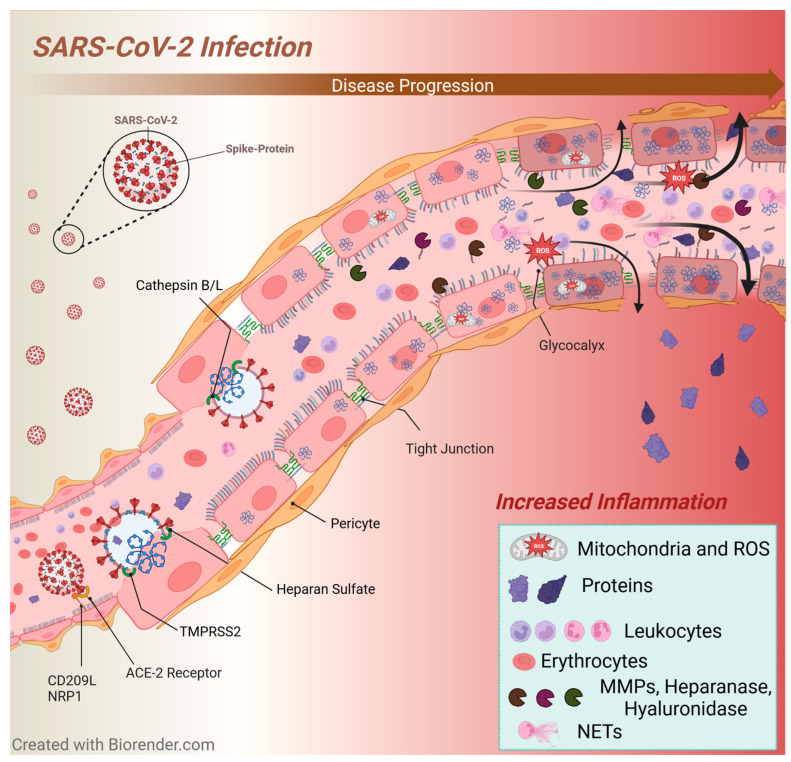
Inflammation during SARS-CoV-2 infection. SARS-CoV-2 enters endothelial cells via the interaction of the spike protein and ACE-2 receptor [236]. Entry is facilitated by CD209L, NRP1, TMPRSS2, heparan sulphate, cathepsin B/L and triggers an endothelial inflammatory response [26,189,237,238,239,240,241,250,281,282]. Viral infection upregulates cytokines production (“cytokine storm”) [283], ROS production [284] and enhances the activity of glycocalyx degrading enzymes such as MMPs, heparanases and hyaluronidases [84,85,125,285,286,287]. Hereby the infection leads to endothelial cell injury, dysfunction and contributes to glycocalyx breakdown [23,24,25,26,189,250,281,282]. Moreover, exposure of the endothelial surface enables immune cells and harmful agents such as ROS to interact more readily with the endothelium [76]. As a result of pro-inflammatory intracellular signalling processes, intercellular junctions are dismantled leading to increased capillary fluid extravasation resulting in oedema formation [93,157,163,164,165,166,167]. Additionally, SARS-CoV-2 might also influence pericytes, which also express ACE-2 [207]. Infection could disrupt vital signalling processes between pericytes and endothelial cells [267,268]. This can stimulate pericyte transdifferentiation into myofibroblast-promoting fibrosis [208], induce pericyte migration [211] and result in wider gaps allowing for greater diapedesis of leukocytes [209,210]. SARS-CoV-2-induced endothelial injury and dysfunction also create a pro-thrombotic state with greater expression of TF, vWF, increased production of thromboxane and endothelin-1, extracellular trap (ET) formation as well as decreased prostacyclin and NO [189,190,191,192,276,288,289,290,291]. Abbreviations: ACE-2 receptor = angiotensin-converting enzyme 2 receptor; CD209L = cluster of differentiation 209; ET = extracellular trap; NETs = neutrophil extracellular traps; MMPs = matrix metalloproteinases; NO = nitric oxide; NRP1 = neuropilin 1; ROS = reactive oxygen species; SARS-CoV-2 = severe acute respiratory syndrome coronavirus 2; TF = tissue factor; TMPRSS2 = transmembrane protease serine 2; vWF = von Willebrand factor.

SARS-CoV-2 can enter host cells utilizing the ACE-2 receptors [236] (Figure 1). Viral entry is facilitated by cluster of differentiation 209 (CD209L), neuropilin 1 (NRP1), transmembrane protease serine 2 (TMPRSS2), heparan sulphate and cathepsin B/L [237,238,239,240,241].

Additionally, the viral S protein is recognized as PAMP (pathogen-associated molecular pattern) by Toll-like receptor (TLR) 2 on monocytes, macrophages, and lung epithelial cells [242].

Virus–cell interactions are suspected to decrease ACE-2 activity and elevate angiotensin II (AngII), a potent vasoconstrictor [243,244,245] that promotes thrombogenicity, oxidative stress and inflammation [236]. The ACE-2 receptor and TMPRSS2 are abundantly expressed throughout the body, found in the respiratory tract and small intestine epithelium, smooth muscle and endothelial cells in arteries, and in cells like pericytes and myocytes [236,246,247]. The wide distribution of ACE-2 and TMPRSS2 expression could explain the variety of symptoms, changes and complications during and after SARS-CoV-2 infection. The virus, in its systemic spread, may attack organs such as the kidneys and heart and can disturb vital organ functions [248].

In the respiratory tract, the virus can cause severe lung injury and breathing difficulties, leading to acute respiratory distress syndrome (ARDS) [249]. For instance, in patients suffering respiratory symptoms, SARS-CoV-2 molecules could be found within capillary endothelial and alveolar epithelial cells, both cell types that express ACE-2 receptors [250,251].

The heart is a major target due to the extensive expression of ACE-2, resulting in potential myocardial damage and cardiovascular complications [248]. SARS-CoV-2 particles have even been discovered in myocardial interstitial cells, either through transient viremia or the migration of infected macrophages, in cases of COVID-19-related cardiogenic shock [252]. Additionally, SARS-CoV-2 antigens were also detected in cardiomyocytes [253].

The gastrointestinal tract is also a major entry and replication site for SARS-CoV-2, supported by high expression of ACE-2 in gastrointestinal cells and the detection of viral RNA in stool samples, which can explain gastrointestinal symptoms such as diarrhoea, vomiting, and abdominal pain [254].

**Figure 2:**
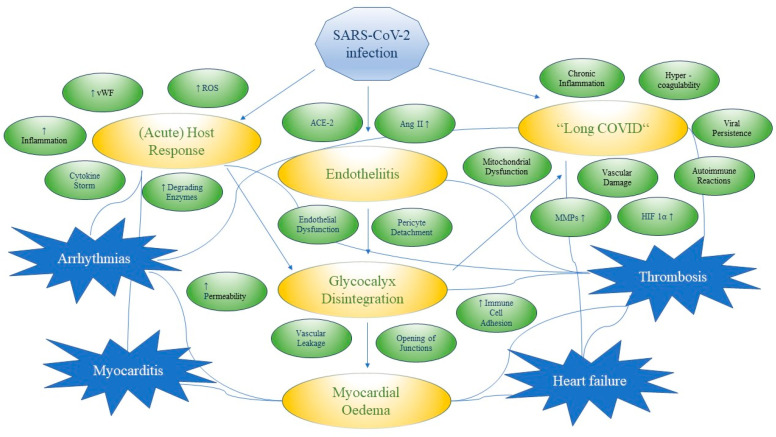
Interrelation between acute and chronic complications of SARS-CoV-2 infection. SARS-CoV-2 infection triggers an unregulated host response, which contributes to glycocalyx disintegration via increased inflammation, greater ROS and cytokine production as well as enhanced activity of glycocalyx degrading enzymes [26,84,85,125,189,237,238,239,240,241,250,281,282,284,285,286,287]. Infiltration of endothelial cells via the ACE-2 receptor triggers endotheliitis, leading to endothelial injury and dysfunction contributing to glycocalyx breakdown [26,189,282]. This leads to greater exposure of the endothelial cell surface area, enabling immune cells and harmful agents to interact more readily with the endothelium [76]. Intercellular junctions are dismantled as a result of the pro-inflammatory intracellular signalling processes, which leads to the breakdown of the endothelial barrier, resulting in enhanced vascular permeability and increased capillary fluid extravasation, culminating in oedema formation [93,157,163,164,165,166,167]. “Long COVID” may stem from mechanisms such as chronic inflammation, mitochondrial dysfunction, (micro-)vascular injury, viral persistence and autoimmune reactions [227,349,350,351,352]. As a result of SARS-CoV-2 infection arrhythmias, myocarditis, thrombosis and heart failure may emerge [292,302,320,321].

SARS-CoV-2 may damage host tissues through a two-phase process, involving rapid virus propagation facilitated by ACE-2 and TMPRSS2, followed by host-specific uncontrolled inflammatory immune responses, leading to collateral tissue damage and potentially systemic failure [255]. The interrelation between acute and chronic complications of SARS-CoV-2 infection are displayed in Figure 2 and discussed in the sections below (see Section 3.5.1, Section 3.5.2, Section 3.5.3, Section 3.5.4 and Section 3.5.5).

Cardiovascular diseases (CVD) themselves have been linked to negative effects on the microvasculature, leading to impaired capillary perfusion and glycocalyx integrity as reported in previous studies [256,257,258,259]. When considering preexisting CVD, it is noteworthy that SARS-COV-2 infection is associated with even more adverse outcomes and greater disease severity [260], as COVID-19 has the potential to exacerbate CVD and evoke microvascular complications [261].

Severe COVID-19 infection can be linked to myocarditis, heart failure, cardiogenic shock, and renal failure [252,262,263,264,265]. These conditions can result in pronounced tissue inflammation, hypoxia, and imbalances in electrolytes, which can contribute to complications such as arrhythmias [266].

SARS-CoV-2 is suggested to interact directly with cardiac cells that have ACE-2 receptors, like pericytes or endothelial cells [247] and impair vascular integrity, leading to damage of the microcirculation which can lead to more inflammation, cardiac fibrosis, and prothrombotic processes [207]. In addition, SARS-CoV-2 infection could enhance microvascular permeability by disrupting signalling processes between pericytes and endothelial cells [267,268]. As a response, pericytes are supposed to release various factors including angiopoietin-2 (Ang-2), transforming growth factor-β1, microRNA-132, and hepatocyte growth factor, which are linked to atrial fibrillation (AF) and promote local tissue inflammation as well as changes in atrial structure and electrophysiology [268,269,270,271,272,273,274,275]. Elevated plasma levels of VEGF, Ang-2, and vWF in patients with AF indicate the contribution to a prothrombotic state [276]. Oedema formation leads to an increase in hydrostatic pressure in heart tissue, which can alter electrical properties and functions [277,278]. Elevated hydrostatic pressure also promotes the risk of AF by enhanced activation of the renin-angiotensin system (RAAS) [279]. This effect has been observed in both human tissues and the atria of hypertensive rats, where the onset of AF coincided with higher levels of angiotensin II, lower levels of angiotensin 1-7 and less ACE-2 receptor activation [279]. In addition, elevated levels of angiotensin themselves can lead to MO [280]. In cardiac tissue, angiotensin II can induce the release of ROS and activate redox-sensitive transcription factors such as NFkB, AP-1, and HIF-1, which stimulates the secretion of VEGF and prostaglandin, ultimately leading to greater vascular permeability [280].

#### 3.5.1. Myocarditis in SARS-CoV-2 Infection

In the context of the COVID-19 pandemic, the incidence, pathophysiology and prevalence of SARS-CoV-2-induced viral myocarditis have been widely discussed. Myocarditis is acknowledged as a possible severe complication of SARS-CoV-2 infection [292]. Moreover, focal fluorodeoxyglucose (FDG) uptake on positron emission tomography (PET) revealed myocardial inflammation even in cases where individuals did not exhibit any cardiac symptoms [293]. Changes on PET corresponded to CMR changes including higher regional T2, T1, and extracellular volume, higher prevalence of LGE, a worse global longitudinal and global circumferential strain and lower left ventricular ejection fraction (LVEF) [293]. The exact mechanisms leading to myocardial injury and potentially myocarditis are yet only poorly understood, but two direct mechanisms (injury by infection of cardiomyocytes and infection of endothelial cells with subsequent endotheliitis) and three indirect mechanisms (hypercoagulability, injury from cytokine storm and systemic inflammation as well as auto-immune mediated damage) are currently under scrutiny [227]. Recent research has suggested that the extremely rare cases of (peri-) myocarditis associated with COVID-19 messenger ribonucleic acid-based vaccine are likely caused by cytotoxic T-cells, pro-fibrotic monocytes, and up-regulation of pro-inflammatory cytokines [294]. However, whether these findings also apply to SARS-CoV-2-induced myocarditis is currently unclear.

The United States Centre for Disease Control calculated the risk of developing myocarditis in the course of COVID-19 to be 0.146% in both in- as well as out-patients, close to 16 times the risk for myocarditis compared to patients without COVID-19 [295]. In one retrospective study involving 6439 patients hospitalized for COVID-19, 37 (0.6%) developed de novo heart failure [296]. Patients recovering from COVID-19 have also reduced cardiac ejection fractions and increased left ventricular volumes compared to healthy controls [297]. Clinical presentations of patients with COVID-19 myocarditis vary from mild to fulminant with concomitant haemodynamic compromise, but most patients make a full clinical recovery [227]. However, CMR revealed that between 30% and 78% of recovered COVID-19 patients show signs of persistent myocardial injury including LGE, prolonged T1 and T2 times indicative of interstitial fibrosis and MO [298,299], as well as signs of ongoing inflammation [297,300].

The long-term consequences of myocardial injury in the course of COVID-19 have yet to be fully established and ought to be monitored regularly with clinical follow-ups, evaluation of cardiac arrhythmia and optimally repeated CMR [227]. A recent study suggests that myocardial injury, indicated by elevated hs-cTnI (>99th percentile) during COVID-19 hospitalization is linked to increased post-discharge mortality and major adverse cardiovascular and cerebrovascular events, underscoring its prognostic importance in COVID-19 survivors [301].

#### 3.5.2. Arrhythmias in SARS-CoV-2 Infection

Arrhythmias, conduction disturbances and changes in the ST segment are reported in more than half of COVID-19 patients three and six months after discharge from hospital [302]. Especially noteworthy is the potential association between SARS-CoV-2 infection and AF, both during the acute infection phase and potentially during recovery [303,304]. COVID-19 is linked to an increased incidence of AF and a detrimental impact on outcomes of hospitalized patients [305]. The reported frequency of arrhythmias and AF in the context of SARS-COV-2 infection and hospitalization varies, with studies showing prevalence rates ranging from 16.7% in Wuhan, China [306], compared to 27.5% of COVID-19 patients admitted to the ICUs in the USA [307].

As the aetiology of electrophysiological changes, different pathomechanisms are discussed. Electrical instability and remodelling mediated by SARS-COV-2 might be a result of mitochondrial dysfunction resulting in a cellular energy deficit as well as oxidative stress [308,309,310]. SARS-COV-2 can directly affect cellular functions by binding to the ACE-2 receptors on cardiomyocytes, cardiofibroblasts, pericytes and endothelial cells [207,311]. Immunohistochemical detection of SARS-CoV-2 spikes and nucleocapsid protein in cardiac atrioventricular nodes has been reported in a patient with lethal COVID-19 infection, suggesting direct interaction with the cardiac conduction system [312]. In addition, the existence of the angiotensin-(1-7)/Mas receptor axis has been reported in rat sinoatrial cells [313]. This pathway is responsible for anti-inflammatory, anti-fibrotic and anti-hypertrophic signalling, a reduction in ROS formation and protection against arrhythmias during reperfusion injury [311,313]. Dysregulation can be observed during acute and possibly also chronic COVID-19 [314,315,316].

Moreover, myocardial oedema formation due to endothelial barrier disruption with activated pericytes is discussed as a major pathomechanism during SARS-CoV-2 infection [317]. The rise in hydrostatic pressures reduces the peak current density of the L-type calcium current and upregulates the transient outward K+ current as well as the ultra-rapid delayed rectifier K+ current, resulting in a shortened action potential duration [278,279]. 

Though not yet proven specifically for COVID-19, the occurrence of arrhythmias has been associated with LGE in CMR, as seen in a relevant proportion of recovered COVID-19 patients [318].

#### 3.5.3. Glycocalyx Changes and Inflammation in SARS-CoV-2 Infection

Recent studies established the role of SARS-CoV-2-induced injury to the vasculature [26,189,250,281,282]. The invasion of SARS-CoV-2 into cells triggers endotheliitis, inducing endothelial damage and dysfunction [26,189,282]. A key part of the COVID-19 disease process driven by SARS-CoV-2 is glycocalyx disintegration, resulting in endothelial dysfunction [23,24,25,26]. This, combined with an unregulated host response that involves both proinflammatory and prothrombotic pathways, significantly contributes to the development of severe COVID-19 [26]. As a consequence, microvascular dysfunction leading to (micro-) thrombosis occurs, which may even result in potentially lethal ischemic multiorgan failure [319,320,321].

Infection with COVID-19 can induce a hyperinflammatory state and unleash a so-called “cytokine storm”, leading to excessive production of various inflammatory mediators including numerous cytokines and chemokines [283]. Overproduction of these substances, including IL-1α, IL-1β, IL-2, IL-7, IL-6, IL-8, IL-10, TNF, interferon (IFN)-γ, granulocyte colony-stimulating factor (G-CSF), IFN-inducible protein-10, monocyte chemotactic protein-1, macrophage inflammatory protein 1 alpha and CXC- chemokine ligand 10 has been described [189,322,323,324,325,326,327,328]. 

Overexpression can have a multitude of effects as molecular signalling pathways of these factors can be quite complex and involve several cell types and proteins. Additionally, during inflammation, endothelial-derived angiopoietin-2 enhances vascular leakage in response to cytokines and inflammatory mediators such as histamine, bradykinin and VEGF [329]. Overexpression of pro-inflammatory factors, especially TNF-α and IL-6, have been observed in patients with dilated CMP and are greatly associated with activation of fibroblasts, collagen deposition and cardiac fibrosis [330,331,332]. Increased levels of IL-6, IL-8, and TNF-α have also been linked to worse outcomes in hospitalized COVID-19 patients [328].

These enhanced inflammatory responses promote the disintegration of the endothelial surface layer. Normally, the glycocalyx is in a balance of constant shedding due to flow and degrading enzymes, and renewal via the production of new components [333]. However, during inflammation ROS [284] and the enzymes capable of degrading the glycocalyx such as hyaluronidases [285], heparinases [286] and MMPs [287] exhibit enhanced activity. In addition, there is increased leucocyte adhesion and diapedesis due to endothelial activation and upregulation of cellular adhesion molecules [77]. The impact of inflammation on the glycocalyx in COVID-19 is evident in the extent of damage it incurs and may be associated with disease severity [334]. Patients with more severe COVID-19 infection, requiring ventilation showed a thinner glycocalyx layer with higher levels of circulating glycocalyx components than non-ventilated patients or healthy controls [335,336,337]. This can be explained by the fact that patients with more severe COVID-19 had higher activity levels of MMPs, hyaluronidase or heparanase [337,338].

#### 3.5.4. Immunothrombosis in SARS-CoV-2 Infection

SARS-CoV-2 triggers a potent immunothrombotic response by the above-described mechanisms. Endothelial injury and dysfunction result in simultaneous activation of platelets and the coagulation cascade as well as proinflammatory pathways [23,24,25,26,27]. Consequently, COVID-19 manifests as an extremely pro-thrombotic condition. The highly pro-thrombotic nature of COVID-19 is evident from the finding of microthrombi in the myocardium and widespread thrombosis in pulmonary vessels in COVID-19 patients [250,339]. Furthermore, thrombus formation has been demonstrated in both large and small blood vessels of arteries and veins [189,340].

Endothelial dysfunction results in decreased prostacyclin and NO production, while increasing the production of thromboxane and endothelin-1, which greatly promotes vasoconstriction and thrombosis [289]. In addition, endothelial injury induces the expression of TF, hereby activating the coagulation cascade [191]. Inflammation also promotes the release vWF from endothelial cells, which can remain anchored to the endothelial surface eventually forming vWF-platelet aggregates [288,341,342].

NET formation can also contribute to immunothrombosis during COVID-19 [343]. Inflammation and infection activate neutrophil granulocytes and induce the formation of NETs, which is a defence mechanism against extracellular organisms [344,345]. Activated neutrophils release their granular and nuclear content to eliminate pathogens [344]. NET formation can be induced by the inflammatory response to the virus [189] and is thought to play a key role in the disease course and severity, as patients exhibit higher markers of NETs in comparison to healthy controls and have worse outcomes [290]. Additionally, NET markers like citrullinated histone H3, cell-free DNA and neutrophil elastase are also associated with greater levels of leukocytes, inflammatory cytokines, and in vivo markers of coagulation, fibrinolysis, and endothelial damage [290]. In the context of SARS-CoV-2 infection, NET formation is increased and impairs endothelial function and vascular integrity, thereby worsening patient outcomes [189,290,291].

#### 3.5.5. Possible Long-Term Effects of SARS-CoV-2 Infection

Following recovery from acute SARS-CoV-2 infection, many patients continue to suffer a wide range of symptoms, commonly referred to as “long COVID” [321,346]. Recent research suggests that up to 30% of patients may still experience symptoms nine months after COVID-19 infection [347]. In patients without previous heart disease, 73% reported cardiac symptoms after approximately 100 days of mild SARS-CoV-2 infection related to signs of inflammation and MO in the heart in CMR [348]. Symptoms persisted in 57% of participants after approximately 1 year and were more likely in females and those with initial widespread heart inflammation [348]. Furthermore, persistent cardiovascular effects due to COVID-19 infection, such as myocardial fibrosis and oedema, as well as changes in the microvasculature like capillary damage, capillary blood flow alterations, tissue hypoxia, and inflammation have been described [227,349]. The potential mechanisms involved in explaining these post-recovery phenomena might include viral persistence, interaction with other viruses, the triggering of autoimmune reactions, and vascular damage culminating in chronic inflammation and microthrombosis [350].

Other emerging studies suggest that SARS-CoV-2 induces chronic inflammation, increased ROS production and a dysbalanced metabolic state due to mitochondrial dysfunction, especially in patients with previous suboptimal mitochondrial function and low mitochondrial reserve due to factors like comorbidities or age [351]. In this context, the release of oxidized DNA fragments from damaged mitochondria activating the NLRP3 inflammasome is thought to be a driving force of chronic inflammation [352].

### 3.6. Cardiotrophic Viruses and Myocarditis

While myocarditis can arise from various pathogens, viruses are suspected to be the most common cause [353,354,355]. In addition, viral and postviral myocarditis are prominent factors contributing to both acute and chronic dilated cardiomyopathy [356]. Viral myocarditis is characterized by cardiac inflammation, necrosis and fibrosis with MO as a frequent complication, which can be detected via CMR [357], and is part of the Lake Louise Criteria [214]. In the literature, several other viruses besides SARS-CoV-2, the so-called cardiotrophic viruses, are also associated with myocarditis and cardiomyopathies [358]. Cardiotropic viruses such as parvovirus B19 [358,359] and human herpesvirus-6 [360] are highly prevalent. Human cytomegalovirus [361,362,363], Epstein–Barr virus [364] and enteroviruses, particularly coxsackie viruses B [365], are described as moderately frequent [358]. Other rare cardiotropic viruses include adenovirus [365,366] and hepatitis C virus [367,368,369]. 

These cardiotropic viruses invade the myocardium using distinct host-cellular pathways resulting in endocytosis [358]. Prior to binding to the virus-specific receptor that initiates entry, the majority of viruses interact with glycocalyx components (Table 2) [370]. 

Herein, the negatively charged heparan sulphate proteoglycans are used by a large variety of viruses for attachment [371,372]. However, the intact glycocalyx hinders virus–cell receptor interaction, meaning, that glycocalyx disruption and removal can uncover the needed receptors and facilitate viral entry [372]. For example, the interaction of ACE-2 receptors with the SARS-CoV-2 spike protein is dependent on the condition of the glycocalyx [373].

Viral entry can trigger a wide range of host-cell responses and activate the innate immune system, as viral proteins can be recognized by TLR1, TLR2, TLR4, TLR6, and TLR10 [374]. For instance, the spike protein of SARS-CoV-2 showed significant binding to TLR1, TLR6 and especially TLR4 [374,375]. Activation of TLR4 can cause cardiac injury and has been linked to cardiac hypertrophy, fibrosis and apoptosis; however, the exact mechanism remains elusive [376]. Active viral replication and its transcription products have been shown to directly cause cardiomyocyte damage or apoptosis in coxsackievirus B3 and adenovirus infection [377,378]. In addition, parvovirus B19 and human herpesvirus 6 can cause vascular endothelial dysfunction, resulting in poor cardiomyocyte function [360,379,380]. Furthermore, myocarditis may occur, when infected immune cells introduce viral genetic material into the myocardial tissue, with viruses such as human herpesvirus 6 and Epstein–Barr virus playing a role [381]. Viral entry follows an inflammatory phase driven by immunological activation, primarily involving natural killer cells and macrophages, leading to cytokine-triggered inflammation, including IL-1, IL-2, TNF, and INF-gamma [382,383]. Initial cardiac damage facilitates the release of inflammatory cytokines and damage-associated molecular patterns (DAMPs) [358,384] initiating the infiltration of mononuclear cells such as lymphocytes and monocytes [385]. The most common inflammatory infiltrate in proven myocarditis is lymphocytic, followed by borderline, granulomatous, giant cell, and eosinophilic [386]. Ultimately, CD4+ and clonal B cell activation sustain local inflammation, leading to further myocyte necrosis, myocardial dysfunction, and additional negative inotropy, as TNF-mediated activation of endothelial cells amplifies cytokine production and inflammation, including inducible nitric oxide synthase activation [387,388]. The increase in proinflammatory chemokines and cytokines activates cardiac myofibroblasts and increases fibrous tissue formation [330]. If the virus persists, viral myocarditis can play a role in the gradual decline in cardiac function and the development of dilated cardiomyopathy [389]. Viral persistence is also associated with a progressive worsening of left ventricular ejection fraction, whereas viral elimination is linked to improved patient outcome as shown for enterovirus-associated cardiomyopathy [390,391]. Active viral replication could play a crucial role in this process [392,393,394]. Viral infections can also induce autoimmune processes leading to chronic inflammation, tissue remodelling, and the development of dilated cardiomyopathy [395,396,397,398,399]. Herein, autoimmune reactions can activate T cells that target the myocardium, potentially based on the cross-reactivity/molecular mimicry phenomenon of viral epitopes and cardiac structures [383,400]. In addition, the production of cytokines like TNF, IL-1a, IL-1b, IL-2 and IFN-γ increases. Cytokines propagate myocardial damage in combination with antibodies against viral and cardiac proteins and, by affecting the contractile apparatus and matrix proteins, worsen systolic heart function [383,401,402,403,404].

**Table 2:** Viruses and interaction with the glycocalyx.

Virus	Glycocalyx Components	Literature Reference
Parvovirus B19	heparan sulphate, sialic acid	[405]
Human herpesvirus 1, 6	heparan sulphate, syndecan-1	[406,407]
Epstein–Barr virus	glycoproteins, hyaluronan synthesis	[408,409]
Human cytomegalovirus	heparan sulphate	[410,411]
Enteroviruses	heparan sulphate, P-selectin glycoprotein ligand-1, sialylated glycan	[412,413,414]
Adenovirus	heparan sulphate, sialic acid	[415,416,417]
Hepatitis C virus	heparan sulphate, syndecan-1	[418,419]
SARS-CoV-2	heparan sulphate proteoglycans	[241,420]
Influenza virus	sialic acids, hyaluronan synthesis	[415,421]

## 4. Clinical Implications and Treatment Options

In addition to established evidence-driven therapeutic guidelines, novel translational approaches to cardiac inflammatory processes are needed to improve outcomes and ameliorate associated complications in these patients. In the context of multiple underlying pathogenic mechanisms contributing to myocardial oedema, a multitargeted therapeutic approach, akin to ancillary therapeutic strategies targeting microvascular obstruction and infarct size reduction in acute coronary syndromes holds promise for potential benefits [422]. This comprehensive approach should consider MO, HF, myocarditis, endothelial dysfunction, glycocalyx degradation, inflammation, and thrombosis. Tailored to the specific aetiology, this could involve a combination of diuretics and heart failure medication, immunomodulation, antiviral therapy, and the administration of agents that regenerate the glycocalyx. In the following paragraphs, some of these concepts will be discussed.

### 4.1. Myocardial Oedema and Myocarditis

Patients with myocardial inflammation, myocarditis and MO have an increased risk of suffering potentially lethal arrhythmia and sudden cardiac death independent of myocardial dysfunction [423,424,425,426]. Both in ischaemic as well as in non-ischaemic cardiomyopathies, positive LGE is associated with a stark increase in the incidence of arrhythmia [318]. This association is especially pronounced in patients with reduced left ventricular ejection fraction ≤30% (HFrEF) [318]. The cumulative evidence suggests a close correlation of LGE and T1-mapping in CMR with myocardial fibrosis, inflammation and oedema [214,215,424,427,428], which has also been validated by endocardial voltage mapping and histopathologic analysis [215,429]. As inflammation, oedema and the subsequent disturbance of cardiac conduction constitute a substrate for serious arrhythmia [318,426,430,431,432], anti-inflammatory treatment strategies may help to alleviate the burden of arrhythmia in addition to guideline-directed medical (GDM) therapy (e.g., anticoagulation, antiarrhythmics). Upon persistence of AF or bradycardia/VT, electrical CV or transient/permanent device therapy may be required [433,434].

In addition to (critical) arrhythmia, myocarditis may also promote heart failure and cardiogenic shock [224,435,436]. In these patients, GDM HF treatment is recommended [224]. In the acute phase characterized by prominent congestion and fluid overload, diuretic treatment with loop diuretics is essential to remove excess water [224]. In severe cases of cardiogenic shock, inotropes and vasopressors, as well as mechanical circulatory support (MCS) such as veno-arterial extra-corporal membrane oxygenation (VA-ECMO), have to be considered [437,438].

Another frequent factor aggravating the inflammatory processes is anaemia, which accounts for worse clinical outcome in hospitalized COVID-19 patients [439,440]. Anaemia leads to enhanced platelet reactivity [441,442] and by contribution to prothrombotic processes and ischaemia, its impact on myocardial oedema formation may be assumed. Therefore, prevention of anaemia should be a cornerstone in primary prevention.

In order to improve cardiac function and alleviate excessive inflammation in acute myocarditis, immuno-modulatory anti-cytokine therapy utilizing targeted biologics may prove viable depending on aetiology [443,444,445,446]. However, it should be noted that—though there were no safety concerns—IL-1ß’s inhibition by canakinumab did not show clinical improvement in the Canakinumab in the COVID-19 Cardiac Injury trial at day 14 [447]. This was a randomized controlled trial comparing canakinumab (at a dose of 600 mg or 300 mg) to placebo in 45 patients with myocardial injury [447]. Future studies regarding dosing regimens and longer follow-up periods are required for canakinumab and other immunotherapies [447,448,449].

In the context of auto-immune mediated myocarditis (e.g., giant cell myocarditis or systemic autoimmune diseases including sarcoidosis, systemic lupus erythematosus (SLE), thyrotoxicosis, or granulomatosis with polyangiitis), corticosteroids, azathioprine, and cyclosporine are utilized [443,446]. However, evidence for these treatment strategies is often weak and recommendations are solely based on expert consensus and pathophysiologic considerations [443,450].

In a retrospective cohort of biopsy-proven virus-negative chronic inflammatory cardiomyopathy, immunosuppression on top of heart failure therapy resulted in improved survival compared to standard heart failure treatment alone [451]. In patients with ventricular arrhythmia, there are some signals that immunosuppression may alleviate arrythmia burden, but evidence remains tenuous [426,434,452].

Experimental and clinical evidence suggests a decisive role for inflammatory cytokines in the regulation of cardiac remodelling, as biomarkers of inflammation are elevated in heart failure patients, even more so during acute cardiac decompensation [453,454,455]. In heart failure associated with diseases other than myocarditis, immunomodulation has failed to demonstrate significant positive effects on mortality [456].

The targeted biologic immunomodulators etanercept and infliximab, both inhibiting TNF-α, have been tested in HFrEF but provided no clinical benefit [457,458]. Left ventricular ejection fraction and left ventricular end-diastolic diameter were improved in a meta-analysis of 19 randomized controlled trials of 1341 patients, therefore indicating a potential role for immunomodulation in HFrEF [456]. In heart failure with preserved ejection fraction (HFpEF), a relevant role for inflammation in the pathogenesis is also assumed, but effective translational therapeutic approaches remain to be explored [453,454,459].

As previously mentioned, CMR is the gold standard for the assessment of myocardial inflammation, oedema and fibrosis [214]. Hence, CMR may also be utilized to guide immunomodulatory therapy in chronic myocarditis [443]. Especially in the context of systemic inflammatory disease, a combination of CMR with functional imaging including fluorodeoxyglucose positron emission tomography could improve diagnosis, management and tapering regimes of immunosuppressive agents [443,460,461,462,463].

Decongestion with diuretics and standard HF treatment also relieves MO [50]. Recent guidelines recommend sodium-glucose cotransporter 2 inhibitors (SGLT-2i) in heart failure patients to reduce the risk for hospitalisation and cardiovascular death [224,464,465]. Though SGLT-2i are suggested to have anti-inflammatory properties and counteract myocardial fibrosis, an improvement of patient survival during hospitalised COVID-19 infection could hitherto not be shown in a meta-analysis including the DARE-19, RECOVERY and ACTIV-4A trial [107,466,467,468,469,470]. Another drug class recommended for heart failure treatment are mineralocorticoid receptor antagonists [224,465]. Herein, finerenone is suggested to preserve endothelial glycocalyx and to protect against COVID-19-associated adverse events in patients with type 2 diabetes and chronic kidney disease [471].

Additional future treatment options could target stimulation of lymphatic water removal. An experimental increase in lymphangiogenesis in a rat model of heart failure post-myocardial infarction has been shown to improve the restoration of myocardial fluid balance, and reduce cardiac inflammation, fibrosis, and dysfunction [472]. Also targeting intravascular pressure, colloid osmotic pressure and intravascular permeability could prove useful.

In myocarditis due to viral infection, direct antiviral therapy, interferon, and intravenous immunoglobulins may be considered depending on the viral pathogen [424]. In chronic viral myocarditis with entero-, or adenovirus, interferon-β treatment increased viral clearance of entero-, adeno-, and parvovirus B19 and reduced endothelial damage in parvovirus B19 infection [365,424,473,474].

Rather than direct viral infection of cardiomyocytes, which has only been demonstrated for enterovirus (e.g., coxsackievirus), molecular mimicry and subsequent auto-immune reaction are thought to contribute to cardiomyocyte injury [424,475,476,477]. However, the therapeutic implications of these findings remain a matter of debate as evidence is currently scarce and randomized controlled trials would be required [424,450].

Currently, several specific antiviral agents are available [424]. Pocapavir and pleconaril as well as intravenous immunoglobulin therapy have been explored for neonatal enteroviral myocarditis [478,479,480]. Anti-herpes virus drugs such as ganciclovir can be used against persistent Epstein–Barr virus, cytomegalovirus or human herpesvirus 6 to reduce viral load [481]. Antiviral therapy against hepatitis C virus-associated myocarditis consists of established antiviral drugs such as ombitasvir, paritaprevir, ritonavir and dasabuvir [482]. Influenza-positive myocarditis can be treated with the neuraminidase inhibitors peramivir and zanamivir [483,484]. Intravenous immunoglobulin therapy is often used in parvovirus B19 infection, with new treatment strategies such as synthetic nucleotide analogues cidofovir and brincidofovir (broad-range antivirals), synthetic coumarin derivates, flavonoid molecules, and hydroxyurea currently being explored [485]. Another approach consists of targeting autoantibodies via immunoadsorption or aptamers (synthetic oligonucleotides that can bind specific molecules like antibodies) [486].

Since SARS-CoV-2-induced COVID-19 is associated with cardiovascular injury, besides direct strategies targeting the virus itself, protection of the endothelium and the glycocalyx, as well as prevention of complications from endothelial injury and dysfunction may prove advantageous [189,226,227,260,261]. Several approaches to combat COVID-19 infection are currently being explored [487]. These include targeting the viral entry mechanisms, immune regulation pathways, or the lifecycle of the virus [487,488].

At the beginning of the COVID-19 pandemic, drug repurposing was explored, but with limited success [488]. Chloroquine and hydroxychloroquine prevent viral entry into the cell via the inhibition of glycosylation of host receptor proteins and manipulation of endosomal proteolytic processing [488]. Furthermore, both agents are also demonstrating anti-inflammatory effects by inhibiting cytokine production by reducing T cell activation [488,489]. Camostat mesylate and arbidol also inhibit host cell entry by inhibiting a host serine protease and interacting with the angiotensin converting enzyme 2 receptor and the S protein, respectively [488]. Lopinavir, darunavir, and remdesivir, agents that interfere with RNA synthesis, were also considered for trial in severe COVID-19 [488].

As of mid-2023, several direct antiviral therapeutics are available [487]. These include inhibitors of RNA-dependent RNA polymerase (remdesivir, molnupiravir, JT001), inhibitors of SARS-CoV-2 main protease (nirmatrelvir, ritonavir, ensitrelvir), and agents, which interfere with the interaction of the S protein and the angiotensin converting enzyme 2 receptor (bebtelovimab, regdanvimab, sotrovimab and others), the latter group being discontinued due to resistance of more recent virus strains [487]. In addition to antiviral agents, in some situations, immunomodulators are also recommended, including glucocorticoids, janus kinase inhibitors, and targeted cytokine antagonists against IL-6 and IL-1β [487].

### 4.2. Endothelial Damage and Glycocalyx Disintegration

In COVID-19, endothelial cell infection leads to dysfunction of the endothelial surface layer and subsequent disturbances of haemostasis, thrombocyte aggregation and MO formation [25,26]. While this may also be observed in early disease stages, endothelial damage is thought to be a major contributor to multi-organ failure in severe COVID-19 [23,24,25,26].

Detailed pathophysiologic insight into the exact processes and deleterious stimuli, to which the endothelium is exposed, both in the context of ischaemia and reperfusion injury as well as viral infection, may inspire several techniques aiming to ameliorate glycocalyx disintegration [113,490,491]. While there are currently no established agents for this indication, various compounds have undergone testing [57].

The administration of nitric oxide during postischemic reperfusion was demonstrated to reduce vascular leakage and vascular resistance, as well as preserve glycocalyx integrity in guinea pig hearts in vitro [492]. Hawthorn extract WSS 1442 has the ability to increase coronary flow by boosting nitric oxide release from vascular endothelium [493], which aligns with safeguarding or augmentation of the glycocalyx [494]. Moreover, WSS 1442 thickens the glycocalyx, which is linked to significantly reduced sodium permeability in vitro [495].

Hyperbaric oxygen, as a preconditioning stimulus, was shown to provide benefits and protection against ischaemia and reperfusion injury [491]. The presumed mechanism involves improving endothelial function and oxygenation while reducing local inflammation, vascular permeability, and tissue oedema [491]. The effect of preconditioning may be appreciated in nuclear magnetic resonance imaging and spectroscopy, where muscle metabolism is positively influenced by preconditioning during reperfusion, with increased production of phosphocreatine and greater oxygen consumption [496].

Furthermore, various agents resembling glycocalyx components are being explored [497,498,499,500,501]. Sulodexide is a natural glycosaminoglycan which regenerates the glycocalyx by boosting glycosaminoglycan synthesis and reducing degradation [497]. In the setting of type 2 diabetes and chronic venous disease, it has been shown to have beneficial effects by regenerating the glycocalyx and combating endothelial dysfunction with anti-inflammatory effects [502,503]. Pentosan polysulphate is an oral heparin-like substance without notable anticoagulant properties and is currently approved by the US Food and Drug Administration for the treatment of interstitial cystitis [504]. Research indicates it boosts glycosaminoglycan levels in diabetic mice and reduces glycocalyx breakdown via decreased MMP activity [57,498]. Wheat germ agglutinin lectin attaches to heparan sulphate and hyaluronic acid and has been shown to decrease albumin filtration and albuminuria in a rat model of chronic kidney dysfunction [499]. Rhamnan sulphate is a heparin-like compound [500] that resulted in an improved glycocalyx and decreased permeability in vitro [505]. Cationic copolymers were designed to specifically boost endothelial barrier function [501]. They have been demonstrated to diminish the increase in hydraulic conductivity caused by shear stress and pressure, as well as to decrease capillary filtration in an isolated perfused mouse lung model, suggesting potential utility in the treatment of pulmonary oedema [501]. As parts of the glycocalyx are often involved in viral entry, this could provide a potential new approach in targeting these components/developing new antibodies targeting these components. For example, interfering with the binding of a virus such as SARS-CoV-2 with heparan sulphate via neutralizing antibodies recovered from COVID-19 patients could be used to combat further infection [241].

Furthermore, substances that interfere with molecular signalling involved in leukocyte diapedesis and migration are also being explored [506]. The inhibition of intercellular adhesion molecule (ICAM)-1 to limit neutrophil infiltration prior to reperfusion exhibited protective properties as it was demonstrated to reduce neutrophil recruitment and smaller infarct size following ischaemia [506].

In a murine model, the extracellular matrix protein Secreted Protein Acidic and Rich in Cysteine (SPARC) was shown to play a crucial role in protecting against cardiac inflammation and mortality in cases of viral myocarditis by preserving the integrity and barrier function of the endothelial glycocalyx [82]. The absence of SPARC leads to increased inflammation, reduced cardiac function, and mortality, but administering recombinant SPARC could reverse these effects, highlighting its potential therapeutic significance in viral myocarditis [82].

Plasma proteins like albumin can be used to treat conditions such as subarachnoid haemorrhage, shock, and trauma [507,508]. In a rodent model of haemorrhagic shock, the degradation of the glycocalyx and subsequent restoration by infusion of plasma was demonstrated in comparison to Ringer lactate [507]. Plasma-treated rodents showed increased syndecan-1 mRNA expression and reduced lung injury. This restoration aligns with S1P effects, where albumin carries S1P that protects against matrix metalloproteinase-mediated glycocalyx degradation [156,509]. Therefore, plasma proteins like S1P could be crucial for safeguarding the glycocalyx. However, the glycocalyx and its implications in various diseases from atherosclerosis to shock and infection remain to be fully understood. Further research in this field is crucial and may provide new insights and novel treatment options.

### 4.3. Thrombosis

Thrombosis in COVID-19 is a critical aspect of disease progression and is closely linked to endothelial dysfunction [189]. As a consequence of glycocalyx degradation and excessive inflammation, platelets are more likely to adhere to the endothelium, provoking both micro- as well as macrovascular thrombosis [27,510,511]. In addition to the degradation of the glycocalyx, TLR-4-dependent mechanisms are also thought to contribute to immunothrombosis in COVID-19 [116,512]. While anticoagulation as a therapeutic measure was accepted by consensus early on in the pandemic, the exact timing, dosage, and duration remain still a matter of debate [513,514,515]. Recent data indicate that the establishment of a therapeutic anticoagulation could potentially improve clinical outcomes in COVID-19 hospitalized patients in non-critical conditions, particularly those at higher risk [516]. However, further research is needed to establish a consensus on antithrombotic therapy in the context of COVID-19 [513,514,515].

## 5. Discussion

Viral infections lead to systemic inflammatory processes affecting (micro-) vascular homeostasis [107,116,321]. During the acute phase, rapid cellular entry is facilitated through the destabilization of the protective endothelial shield, the glycocalyx [321]. The breakdown of the endothelial barrier leads to enhanced permeability shifting inflammatory processes to adjacent tissues [321]. The chronic phase comprises viral persistence and sub-clinical inflammation triggering immunothrombosis, oedema formation, tissue necrosis and, more specifically, myocardial remodelling resulting in heart failure [1,4,107,116,190,291,390,404].

Many viruses contribute to endoplasmic reticular stress by using the cell’s machinery for the production of large amounts of viral proteins as well as double-stranded RNA intermediates as part of their replication cycle [517]. Consequently, dsRNA is recognized by cytosolic pattern recognition receptors (PRRs) triggering pro-inflammatory responses, which include protein kinase R (PKR) and oligoadenylate-ribonuclease L (OAS-RNase L) activation as well as interferon production [518]. PKR activation is also central in the signalling cascade mediated by TLR-4 after sensing of viral glycoproteins [116]. This pathway seems to have a regulatory function while promoting the activation of the transcription factor nuclear factor erythroid-2-related factor 2 (NRF2), which enhances the expression of antioxidant enzymes, such as superoxide dismutase 1 (SOD-1) or heme oxygenase 1 (HO-1) [519,520]. HO-1 induction has been shown to ameliorate reperfusion patterns and reduce ischaemia/reperfusion injury and oedema formation [521,522,523]. 

Immunothrombosis is a crucial pathway during acute SARS-COV-2 infection and is initiated by TLR-signalling [116]. Herein, ET formation results in the release of nuclear as well as mitochondrial DNA and histones. The latter are recognized as DAMPs and promote thrombin generation through TLR-2 and TLR-4 on platelets [524,525]. Thrombin can activate platelets even at subnanomolar concentrations and despite P2Y12 inhibition it still promotes the formation of a considerable and stable amount of platelet aggregates [526,527,528,529,530]. Herein, it could be shown that ticagrelor inhibits thrombin-mediated platelet activation more strongly than prasugrel in patients with acute coronary syndrome and dual antiplatelet therapy [531]. 

(Micro-) thrombotic complications during SARS-CoV-2 infection are furthermore driven by the TLR-vWF-NETosis axis, destabilizing microvascular integrity [116,321]. 

Interestingly, in patients with acute lung injury/acute respiratory distress syndrome, higher angiopoietin-2, as well as vWF levels, were associated with pulmonary permeability oedema [532]. Moreover, oedema formation during SARS-CoV-2 was also described in the brains of COVID-19 patients with neurological symptoms. Herein, widespread white matter volume shifts corresponding to vasogenic oedema were detected by multi-compartment diffusion microstructure imaging sequences in magnetic resonance imaging [533]. These changes together with fibrinogen leakage suggest the disruption of the blood–brain barrier [533,534]. The latter is characterized by a breakdown of pericyte homeostasis and perivascular inflammation, as documented in brain tissue samples from autopsies of COVID-19 patients [534]. SARS-CoV-2 infection of pericytes triggers in consequence viral entry into the central nervous system [534]. Moreover, viral persistence in the central and peripheral nervous system accounts for different post-COVID sequelae including symptoms of cognitive concerns and dysautonomia such as postural orthostatic tachycardia syndrome (POTS) and small fibre neuropathy [535,536,537]. In this context, it is hypothesized that microvascular dysfunction with interruption of pericyte integrity is one of the underlying major causes of post-COVID sequelae [538].

Also in the heart, SARS-COV-2 affects the microvascular barrier including pericytes, and, further, cardiomyocytes and fibroblasts [539,540,541]. In a model of COVID-19 in hamsters, the occurrence of fibrin-rich microthrombi and loss of pericytes was associated with oedematous cardiomyocyte swelling [542]. In addition, the disruption of human cardiac pericytes is caused by the SARS-CoV-2 S protein in an extracellular signal-regulated kinase 1/2 (ERK1/2) dependent manner through the CD147 receptor [543]. However, the phosphorylation of HIF-1 by ERK 1/2 promotes its nuclear accumulation and control of HIF-1 target gene expression. Surprisingly, however, SARS-CoV-2 infection inhibits HIF1α translocation in cultured cardiomyocytes, as well as in an epithelial cell line [542]. Therefore, it can be assumed that local hypoxia, which is conferred by prothrombotic and inflammatory stimuli together with impaired hypoxia resolvability is responsible for cardiomyocyte oedema formation [542]. 

In summary, microvascular homeostasis including glycocalyx and pericyte integrity plays a key role in the maintenance of the endothelial barrier and interstitial fluid balance [77]. The impairment of the endothelial surface layer during SARS-CoV-2 infection due to inflammation and ischaemia significantly enhances vascular permeability resulting in oedema formation [61,66,77,79,542].

Further research on the pathophysiology and treatment concepts is warranted to obtain a more detailed insight into unique signalling pathways and possible therapeutic opportunities during acute and chronic SARS-CoV-2 infection.

## Figures and Tables

**Table 1:** Revised Starling equation and numerical estimates.

JV = LPS[(PC-PI)-σ(ΠC-ΠG)]
L_P_S: ~0.35 mL min^−1^ mmHg^−1^ 100 g^−1^ [31,32]
S: 500 cm^2^ g^−1^ [32,33]
P_C_: end-diastolic 20–30 mmHg [34,35]
P_I_: ~120 mmHg during systole, 15 mmHg during diastole [36,37]
σ: 0.51–0.67 for plasma proteins; 0.41–0.59 for albumin [36,38,39]
Π_C_: 21–24 mmHg [36,40,41]
Π_G_: 13–20 mmHg [42]

J_V_ = Rate of volume filtration. L_P_ = hydraulic conductivity, i.e., permeability or how easily fluid passes through a certain medium/pores. S = surface filtration area, i.e., endothelial area in case of blood vessels. P_C_-P_I_ = intracapillary hydrostatic pressure (P_C_)—interstitial hydrostatic pressure (P_I_). σ = protein reflection coefficient describes the permeability of a membrane to a given solute. Π_C_-Π_G_ = intracapillary colloid osmotic pressure (Π_C_)—subglycocalyx colloid osmotic pressure (Π_G_).

## Abbreviations

ADAM10 (a disintegrin and metalloproteinase 10)
AF (atrial fibrillation)
AJ (adherens junctions)
ANP (A-type natriuretic peptide)
Ang-1 (angiopoietin-1)
Ang-2 (angiopoietin-2)
AngII (angiotensin II)
cAMP (cyclic adenosine monophosphate)
CD39 (ecto-ADPase)
CD209L (cluster of differentiation 209)
CMR (cardiac magnetic resonance imaging)
CNP (C-type natriuretic peptide)
CVD (cardiovascular disease)
DAMPs (damage-associated molecular patterns)
EMB (endomyocardial biopsy)
Epac1 (guanine nucleotide exchange factor 3)
ERK1/2 (extracellular signal-regulated kinase 1/2)
ESL (endothelial surface layer)
ET (extracellular trap)
FDG (fluorodeoxyglucose)
G-CSF (granulocyte colony-stimulating factor)
GDM (guideline directed medical)
GEFs (guanine nucleotide exchange factors)
HFrEF (heart failure with reduced ejection fraction)
HFpEF (heart failure with preserved ejection fraction)
HIF (hypoxia-inducible factor)
HO-1 (heme oxygenase 1)
ICAM (intercellular adhesion molecule)
IFN (interferon)
IL (interleukin)
LDL (low-density lipoprotein)
LGE (late gadolinium enhancement)
LPS (lipopolysaccharide)
LVEF (left ventricular ejection fraction)
mECM (myocardial extracellular matrix)
MCS (mechanical circulatory support)
MMPs (matrix metalloproteinases)
MO (myocardial oedema)
NET (neutrophil extracellular trap)
NO (nitric oxide)
NRF2 (nuclear factor erythroid-2-related factor 2)
NRP1 (neuropilin 1)
NT-proBNP (N-terminal prohormone of BNP)
OAS-RNase L (oligoadenylate-ribonuclease L)
PAMP (pathogen-associated molecular pattern)
PET (positron emission tomography)
PKA (protein kinase A)
PKR (protein kinase R)
POTS (postural orthostatic tachycardia syndrome)
PRRs (pattern recognition receptors)
RAAS (renin-angiotensin-aldosterone system)
ROS (reactive oxygen species)
S (surface filtration area)
S1P (sphingosine-1-phosphate)
SARS-CoV-2 (severe acute respiratory syndrome coronavirus 2)
SGLT-2i (sodium-glucose cotransporter 2 inhibitors)
SPARC (Secreted Protein Acidic and Rich in Cysteine)
SOD-1 (superoxide dismutase 1)
Tiam1 (T-lymphoma invasion and metastasis inducing protein 1)
TF (tissue factor)
TIMPs (tissue inhibitors of metalloproteinases)
TLR (Toll-like receptor)
TNF-α (tumor necrosis factor-α)
TMPRSS2 (transmembrane protease serine 2)
TJ (tight junctions)
Trio (Trio Rho guanine nucleotide exchange factor)
Vav2 (guanine nucleotide exchange factor 2)
VA-ECMO (veno-arterial extra-corporal membrane oxygenation)
VE-cadherin (vascular endothelial-cadherin)
VEGF (vascular endothelial growth factor)
vWF (von Willebrand factor)
ZO (zonula occludens)
α-klotho (alpha-klotho)
Δ P (difference between capillary and interstitial hydrostatic pressure)
Δ Π (difference between osmotic pressure across the membrane derived from plasma proteins)
σ (protein reflection coefficient)
Π_C_ (intracapillary colloid osmotic pressure)
Π_G_ (subglycocalyx colloid osmotic pressure)
P_C_ (intracapillary hydrostatic pressure)
P_I_ (interstitial hydrostatic pressure)
